# MITOCHONDRIAL ENERGETICS IN SKELETAL MUSCLE ARE ASSOCIATED WITH LEG POWER AND CARDIORESPIRATORY FITNESS IN SOMMA

**DOI:** 10.1093/geroni/igae098.0657

**Published:** 2024-12-31

**Authors:** Theresa Mau, Philip Kramer, Paul Coen, Russell Hepple, Stephen Kritchevsky, Anne Newman, Peggy Cawthon, Steven Cummings

**Affiliations:** California Pacific Medical Center, San Francisco, California, United States; Wake Forest University School of Medicine, Winston-Salem, North Carolina, United States; AdventHealth, Orlando, Florida, United States; University of Florida, Gainesville, Florida, United States; Wake Forest University School of Medicine, Wake Forest University School of Medicine, North Carolina, United States; University of Pittsburgh, University of Pittsburgh, Pennsylvania, United States; California Pacific Medical Center, San Francisco, California, United States; California Pacific Medical Center, San Francisco, California, United States

## Abstract

Mitochondrial energetics are an important property of aging muscle, as generation of energy is pivotal to the execution of muscle contraction. However, its association with functional outcomes, including leg power and cardiorespiratory fitness, is largely understudied. In the Study of Muscle, Mobility and Aging, we collected vastus lateralis biopsies from older adults (n = 879, 70–94 years, 59.2% women). Maximal State 3 respiration (Max OXPHOS) was assessed in permeabilized fiber bundles by high-resolution respirometry. Capacity for maximal adenosine triphosphate production (ATPmax) was measured in vivo by 31 P magnetic resonance spectroscopy. Leg extension power was measured with a Keiser press system, and VO2peak was determined using a standardized cardiopulmonary exercise test. Gender-stratified multivariate linear regression models were adjusted for age, race, technician/site, adiposity, and physical activity with beta coefficients expressed per 1-SD increment in the independent variable. Max OXPHOS was associated with leg power for both women (β = 0.12 Watts/kg, p <.001) and men (β = 0.11 Watts/kg, p <.050). ATPmax was associated with leg power for men (β = 0.09 Watts/kg, p <.05) but was not significant for women (β = 0.03 Watts/kg, p =.11).

